# An evaluation of irreversible electroporation thresholds in human prostate cancer and potential correlations to physiological measurements

**DOI:** 10.1063/1.5005828

**Published:** 2017-10-09

**Authors:** Sabrina Campelo, Massimo Valerio, Hashim U. Ahmed, Yipeng Hu, Sara L. Arena, Robert E. Neal, Mark Emberton, Christopher B. Arena

**Affiliations:** 1 Laboratory for Therapeutic Directed Energy, Department of Physics, Elon University, Elon, North Carolina 27244, USA; 2 Division of Surgery and Interventional Science, University College London, London NW1 2BU, United Kingdom; 3 Department of Urology, University College London Hospitals NHS Foundation Trust, London NW1 2PG, United Kingdom; 4 Department of Urology, Centre Hospitalier Universitaire Vaudois, Lausanne 1011, Switzerland; 5 Centre for Medical Image Computing, University College London, London WC1E 6BT, United Kingdom; 6 Department of Exercise Science, High Point University, High Point, North Carolina 27268, USA; 7 Department of Biomedical Engineering and Mechanics, Virginia Tech, Blacksburg, Virginia 24061, USA; 8 AngioDynamics, Marlborough, Massachusetts 01752, USA

## Abstract

Irreversible electroporation (IRE) is an emerging cancer treatment that utilizes non-thermal electric pulses for tumor ablation. The pulses are delivered through minimally invasive needle electrodes inserted into the target tissue and lead to cell death through the creation of nanoscale membrane defects. IRE has been shown to be safe and effective when performed on tumors in the brain, liver, kidneys, pancreas, and prostate that are located near critical blood vessels and nerves. Accurate treatment planning and prediction of the ablation volume require *a priori* knowledge of the tissue-specific electric field threshold for cell death. This study addresses the challenge of defining an electric field threshold for human prostate cancer tissue. Three-dimensional reconstructions of the ablation volumes were created from one week post-treatment magnetic resonance imaging (MRIs) of ten patients who completed a clinical trial. The ablation volumes were incorporated into a finite element modeling software that was used to simulate patient-specific treatments, and the electric field threshold was calculated by matching the ablation volume to the field contour encompassing the equivalent volume. Solutions were obtained for static tissue electrical properties and dynamic properties that accounted for electroporation. According to the dynamic model, the electric field threshold was 506 ± 66 V/cm. Additionally, a potentially strong correlation (*r* = −0.624) was discovered between the electric field threshold and pre-treatment prostate-specific antigen levels, which needs to be validated in higher enrollment studies. Taken together, these findings can be used to guide the development of future IRE protocols.

## I. INTRODUCTION

Irreversible electroporation (IRE) is a non-thermal, soft tissue ablation modality^1^ that has been used to treat a variety of tumors in the liver,^2^ kidneys,^3^ pancreas,^4,5^ and prostate.^6–9^ This technique involves generating pulsed electric fields between electrodes that are inserted into or around the region of interest. If the electric field is of sufficient strength and duration, cells die through the creation of long-lasting nanopores in the plasma membrane.^10^ Because the mechanism of cell death does not rely on extreme temperatures, IRE can be performed safely near major blood vessels^11^ and nerves.^12^ Therefore, it is gaining momentum as a viable treatment option for surgically inoperable tumors and an alternative to functionally limiting resection procedures.

The first human IRE trials in the prostate were performed by Onik and Rubinsky.^13^ Following the placement of 4 electrodes in a square grid, a NanoKnife^®^ generator was used to apply IRE pulses between all pairs, including the diagonals (six treatments). Immediately after treatment, continence and potency were preserved, even when ablations involved the urethra and ejaculatory ducts. Additionally, biopsies taken within the treatment zone showed no evidence of cancer and exhibited characteristics encountered in previous animal studies, including a well-demarcated ablation and vascular and nervous preservation.^14,15^

Recent clinical trials have demonstrated similar levels of success in a two-center study by Valerio *et al.* after a median follow-up of six months.^7^ Specifically, 75% (18/24) of patients exhibited no signs of residual disease, 100% (24/24) were continent, and potency was preserved in 95% (19/20) of those potent before treatment. Also, no patients had a recto-urethral fistula or urethral stricture. Prospective studies with higher enrollment are currently underway to confirm efficacy.^8,9,16^

Despite the initial encouraging results, relatively little is known about the electrical response of prostatic tissue to IRE. For a given set of pulse parameters (pulse duration, number, and repetition rate), the electric field, which is controlled by the applied voltage and electrode spacing, is the primary factor in defining the spatial distribution of cell death. The macroscopic electric field controls the microscopic increase in transmembrane potential (TMP) and the induction of nanopores. The electric field threshold for cell death has been characterized in several tissue types, including normal porcine liver (423 V/cm),^17^ normal canine kidneys (575 V/cm),^18^ normal canine brain (495–510 V/cm),^19^ normal canine prostate (948 V/cm),^20^ and normal human prostate (1135 V/cm).^20^ Additionally, Neal *et al.* determined a dynamic conductivity function specific to normal canine prostate based on intrapulse voltage and current measurements.^20^

A significant challenge remains in defining the electric field threshold for human prostate tumors. Here, we utilize contrast-enhanced magnetic resonance imaging (MRI) data from a human clinical trial^7^ to reconstruct ablation volumes and predict the electric field threshold. Specifically, a numerical model was created for the electrodes embedded within the ablation volume, and the dynamic conductivity function was varied until the calculated current matched the current measured during the clinical trial. Then, the field threshold was determined by matching the ablation volume to the volume encompassed by a specific electric field contour. Our results indicate that the average electric field threshold predicted by the dynamic model was 506 V/cm. Additionally, we found a correlation (*r* = −0.624) between the field threshold and pre-treatment prostate-specific antigen (PSA) levels. Taken together, this information could be used to improve the accuracy of IRE treatment planning algorithms for prostate cancer.

## II. RESULTS AND DISCUSSION

**FIG. 1.**
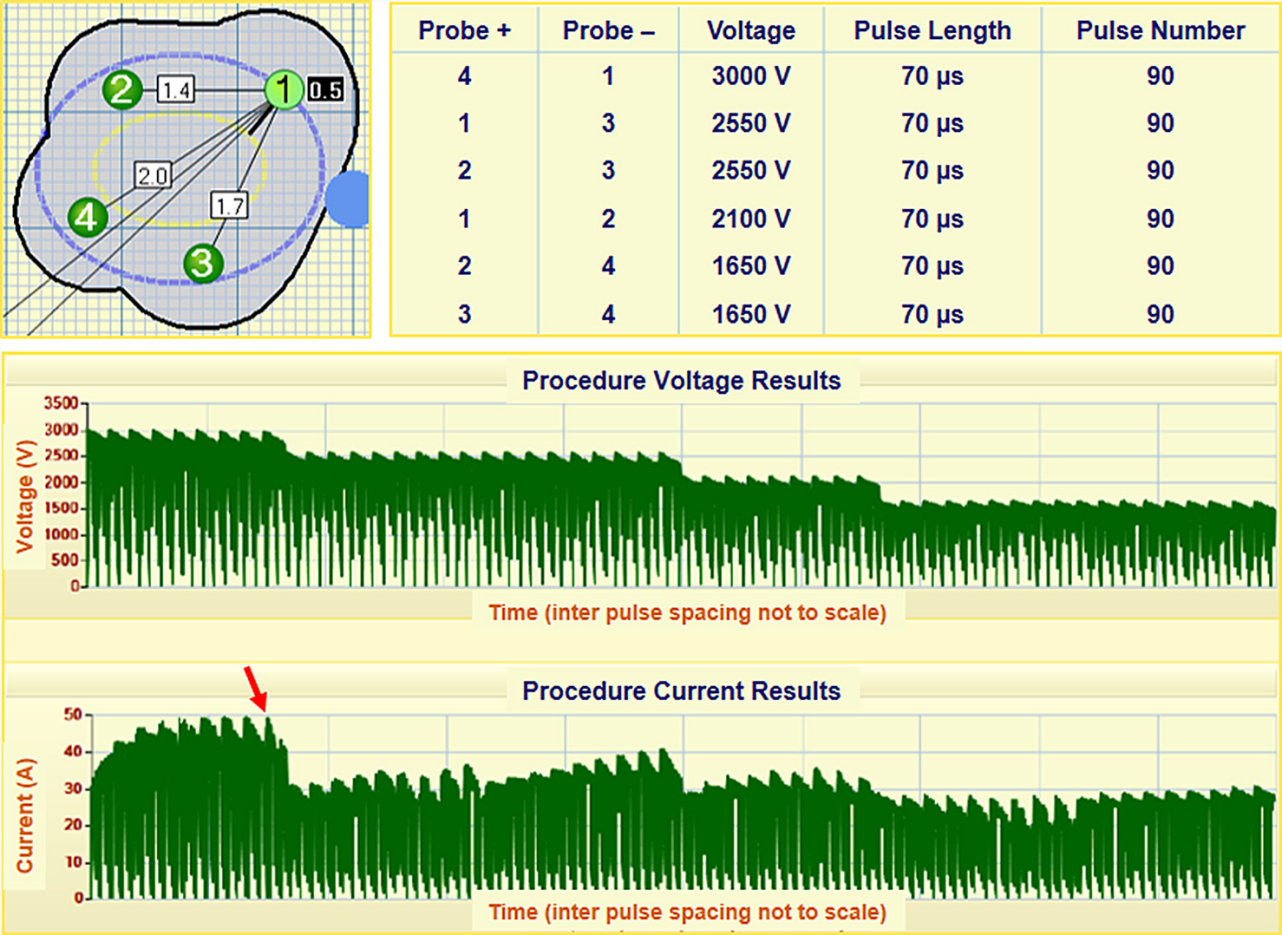
Electrode positioning and pulse protocol for patient P2. The graphical user interface calculates the inter-electrode spacing and the necessary voltage to achieve 1500 V/cm. Voltage and current are reported following treatment. The NanoKnife delivers pulses in groups of 10 before recharging the capacitor bank, as evident by the periodic drop in voltage and current. The red arrow indicates the current matched during the derivation of the conductivity functions.

**FIG. 2.**
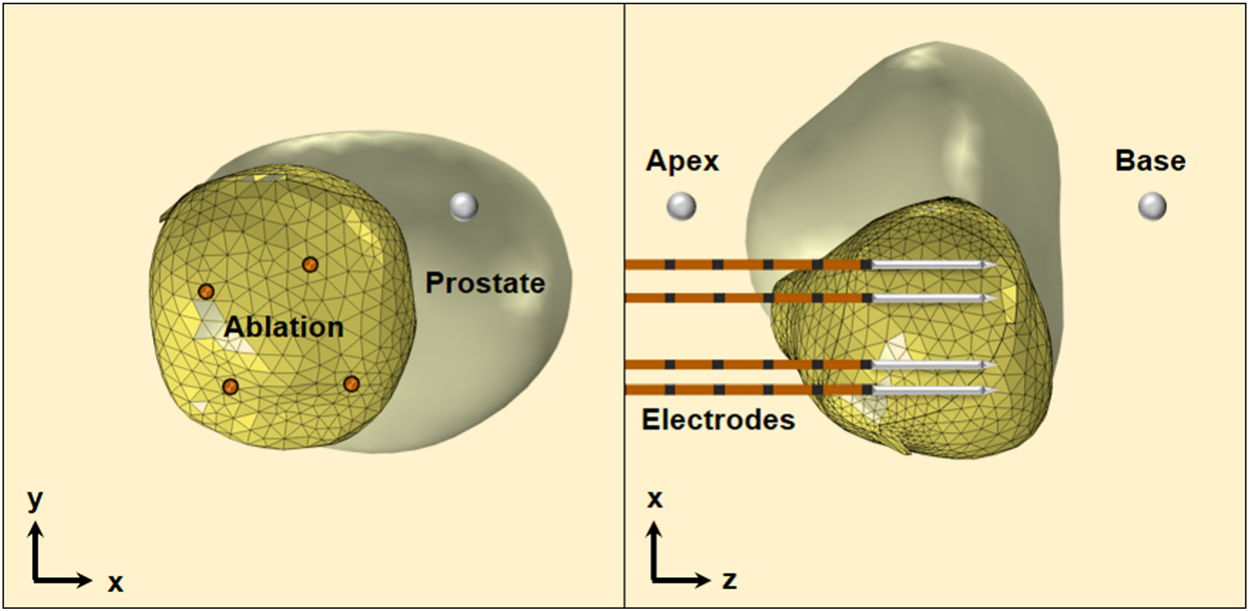
IRE electrode placement in the 3D reconstruction of the ablation volume for patient P2. Only the ablation volume was utilized in the finite element simulations. Additionally, the electrodes were treated as cylinders with dimensions dictated by the exposure length, and the insulation and sharp tip were excluded from the simulations.

Treatment of prostate cancer involves a variety of strategies ranging from active surveillance to radical prostatectomy. Focal therapy with IRE may serve an excellent medium by achieving local oncological control with prostatic tissue preservation. Further, patient morbidity may be reduced, and functional outcomes may be improved compared to other techniques, such as brachytherapy, cryoablation, and high-intensity focused ultrasound, whose results have been summarized in Ref. 21. Refer to Sec. IV for a description of the steps for treating patients using a NanoKnife^®^ (Fig. 1) and simulating the treatments using finite element modeling (Fig. 2).

Determining the electric field threshold for IRE is a vital step in the treatment planning process. It ensures complete destruction of the targeted zone while preserving as much of the healthy tissues as possible. In practice, this improves patient outcomes. Our results indicate that the IRE threshold ranges from 412 to 614 V/cm (Table I) with an average of 506 ± 66 V/cm. These values were obtained using the dynamic conductivity function. For the static model, the IRE threshold ranges from 277 to 573 V/cm with an average of 422 ± 90 V/cm.

**TABLE I.** Treatment characteristics of patients.

Patient No.	PSA Pre-IRE	Max voltage (V)	Max spacing (cm)	Change in current (A)	Electric field threshold (dynamic model) (V/cm)	Ablation volume (cm^3^)
P1	10.03	3000	2.2	11	412	21.08
P2	9.1	3000	2.0	19	436	16.73
P3	8.7	3000	2.1	12	521	11.10
P4	7.8	2850	1.9	11	499	15.82
P5	7.1	2850	1.9	11	512	10.34
P6	6.7	3000	2.0	10	472	19.07
P7	6.5	2700	1.8	10	525	10.05
P8	5.5	2250	1.5	10	614	4.09
P9	3.9	2400	1.6	9	467	10.21
P10	3.8	2100	1.4	6	606	4.63

**FIG. 3.**
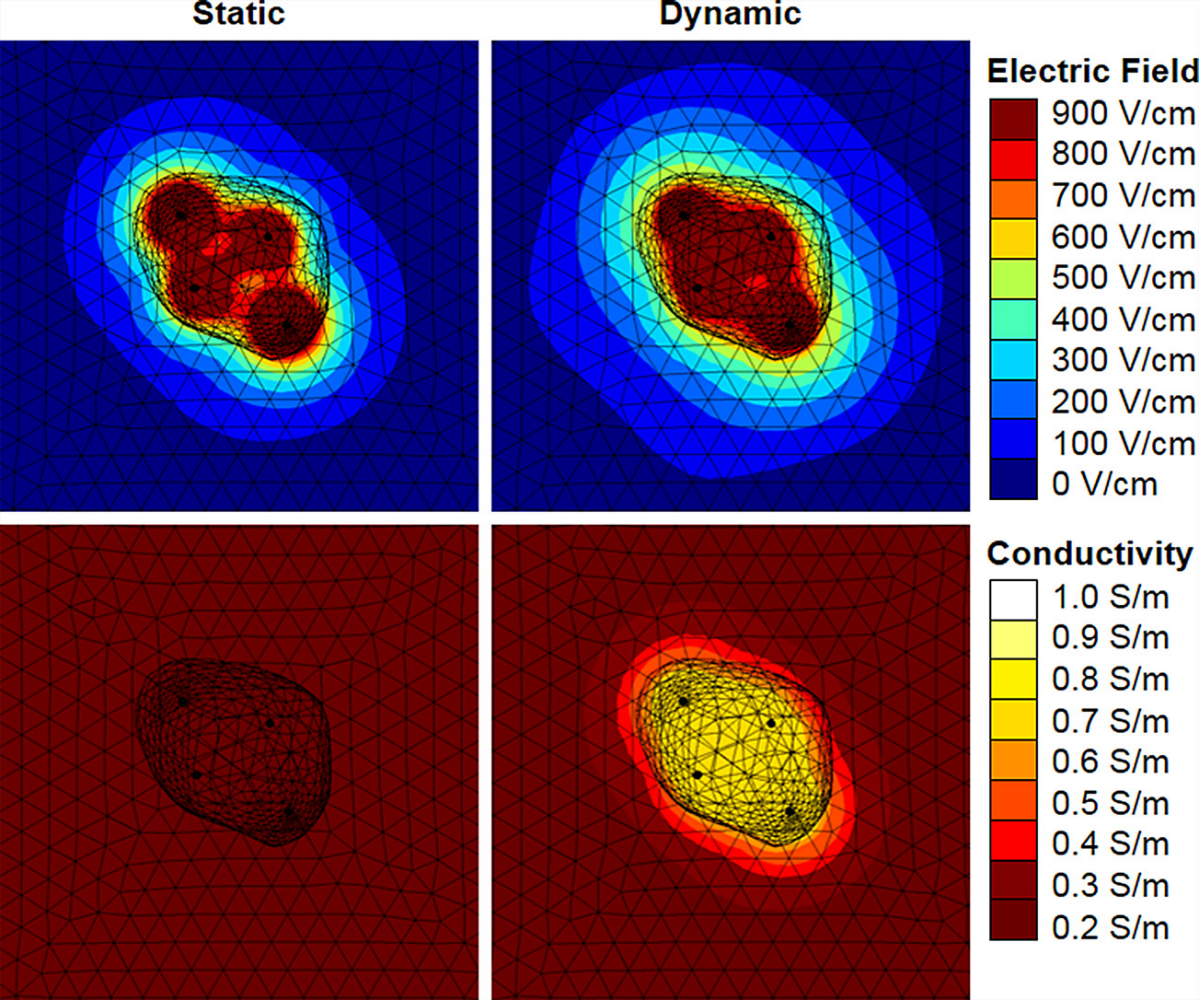
Maximum electric field (top) and electric conductivity (bottom) achieved during six pulsing sequences between all electrode pair combinations for patient P3. The results for static electric conductivity are shown in the left two panes, and the results for dynamic electric conductivity due to electroporation are shown in the right two panes.

**FIG. 4.**
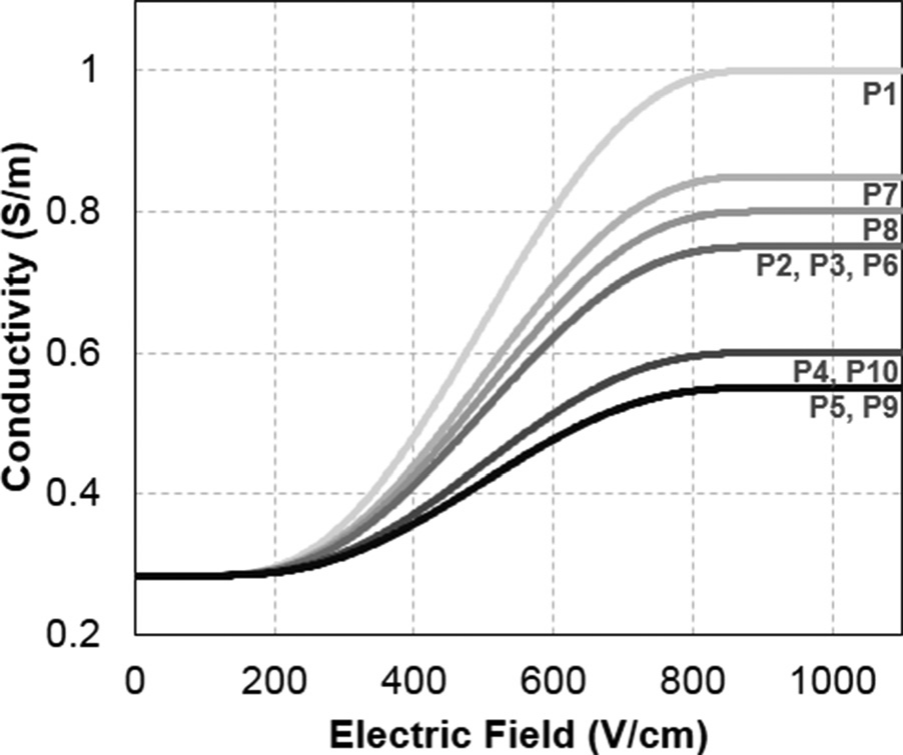
Dynamic conductivity function for electroporation utilized in each patient-specific simulation. The height of the step function was determined parametrically until the calculated current matched the experimental current delivered by the NanoKnife.

Representative electric field and conductivity distributions are shown for patient P3 in Fig. 3. The morphology of the electric field contours for both the static and dynamic models closely resembles the shape of the ablation volume. However, in the dynamic model, the influence of electroporation causes the electric field to spread away from the electrodes. This explains the higher IRE thresholds observed in the dynamic model. The underlying conductivity functions for all patients are shown in Fig. 4. The step height (*σ*_max_) ranges from 0.55 to 1.0 S/m with an average of 0.72 ± 0.15 V/cm.

It should be noted that the electric field thresholds determined for the clinical protocols utilized here in cancerous tissue (506 V/cm) are significantly lower than those previously reported in benign prostate parenchyma (1135 V/cm, an average of two trials).^20^ In addition to the tissue type, this discrepancy is likely due to a number of compounding factors pertinent to the studies. The thresholds in Ref. 20 were derived from numerical simulations calibrated to pathologic lesions three and four weeks post-IRE versus radiologic lesions one week post-IRE. The additional time between the IRE treatment and lesion evaluation permits a significant lesion resolution,^14^ which would require calibrating the electric field threshold to a smaller volume, resulting in a significantly higher threshold. Furthermore, Ref. 20 used a two-needle electrode array with a single treatment, and the lesions calibrated in this study used a four-needle electrode array with six total treatments. The overlap between successive treatments likely reduced the electric field threshold for cell death, as those regions experienced an elevated number of pulses.^22^

Statistical correlations between IRE parameters and PSA measures are summarized in Table II. Pre-treatment PSA demonstrated a potentially strong correlation with the electric field threshold (*r* = −0.624, *p* = 0.054). Even though this relationship did not reach statistical significance, the analysis was under-powered, most likely due to the sample size (Table II). Pre-treatment PSA demonstrated a strong significant relationship with the change in the current (*r* = 0.694; *p* = 0.026) and ablation volume (*r* = 0.730; *p* = 0.017). Additionally, the change in PSA demonstrated a strong significant relationship with the ablation volume (*r* = 0.843; *p* = 0.004), and the electric field threshold demonstrated a strong significant relationship with the ablation volume (*r* = −0.896; *p* = <0.001). The latter may be due to larger regions of overlap between successive treatments.

**TABLE II.** Correlations and *p*-values.

		*r* (*p*)	*Power (%)*
Pre-treatment PSA	Electric field threshold	−0.624 (0.054)	50.9
Pre-treatment PSA	Change in current	0.694 (0.026)^a^	66.6
Pre-treatment PSA	Ablation volume	0.730 (0.017)^a^	75.4
Change in PSA	Ablation volume	0.843 (0.004)^a^	94.2
Electric field threshold	Ablation volume	−0.896 (<0.001)^a^	99.9

^a^ Statistical significance *p* < 0.05.

**FIG. 5.**
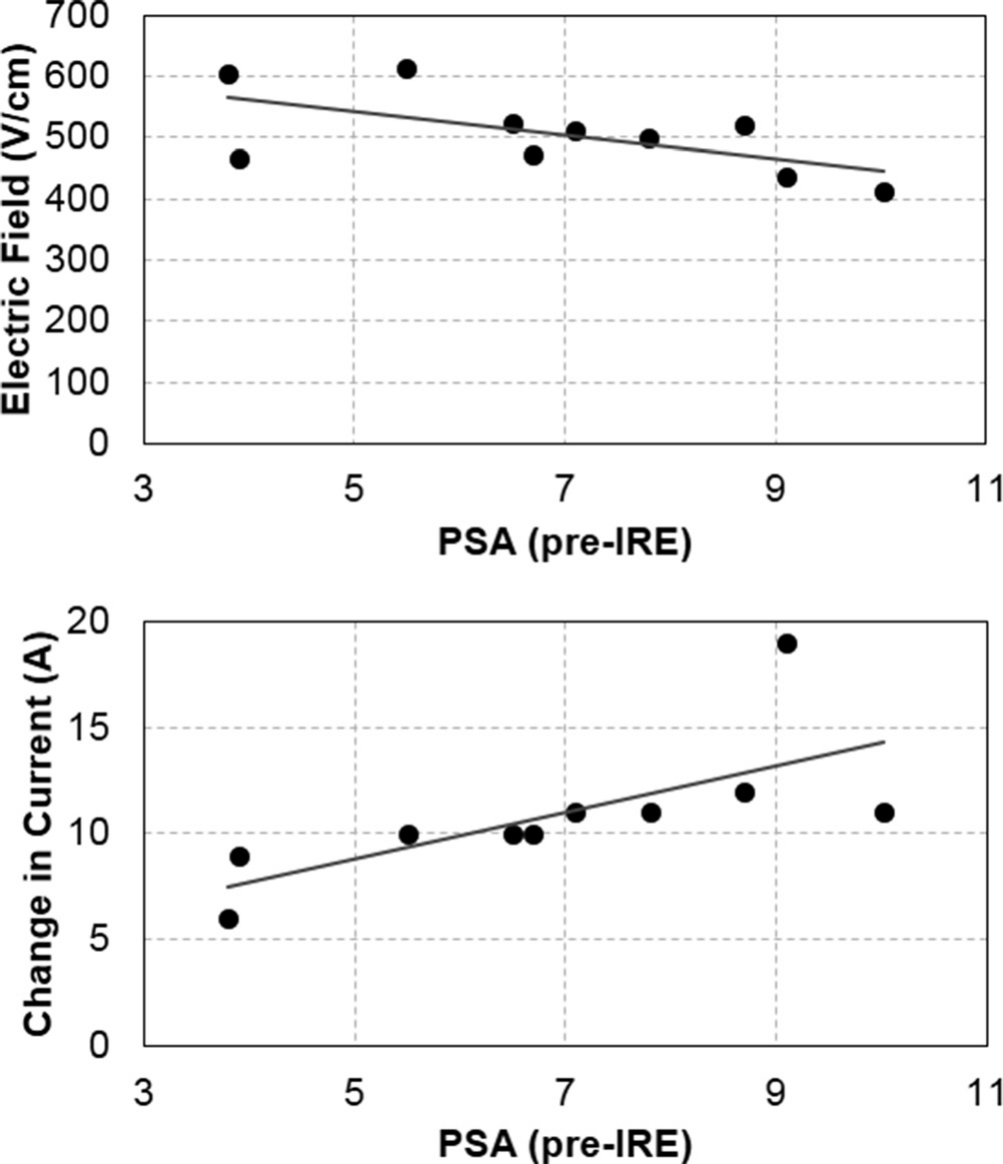
(Top) Correlation between the pre-treatment PSA and the electric field threshold (*r* = −0.624; *p* = 0.054). (Bottom) Correlation between the pre-treatment PSA and the change in current (*r* = 0.694; *p* = 0.026).

Perhaps, the two most interesting correlations are between the pre-treatment PSA and the electric field threshold and between the pre-treatment PSA and the change in current (Fig. 5). Being able to modify the aggressiveness of the treatment protocol according to *a priori* knowledge of PSA safeguards against under-treatment, which can lead to recurrence. One possible explanation for the strong (yet insignificant) correlation between PSA and IRE thresholds is related to the cell size. According to the Schwan equation^23^

$${\;\text{TMP}}_{max}=\;1.5\;E\;r,$$

where the maximum transmembrane potential (TMP) across a spherical cell is a function of the applied electric field (*E*) and cell radius (*r*). Larger cells are able to reach a critical TMP for IRE (e.g., 1 V) with a lower electric field (i.e., larger cells have a lower IRE threshold). Previous studies have shown that the PSA level is correlated strongly with tumor diagnosis and aggressiveness.^24^ As malignancy is multifactorial, it is not possible to generalize that more aggressive tumors are comprised of larger cells. Oftentimes, it depends on the type of tumor, and the prostate literature focuses on links between the cancer progression and Gleason score,^25^ nuclear morphology,^25^ and centrosome size.^26^ The well-studied DU145 and PC3 cell lines do show a dependence of the cell size on aggressiveness, with the larger PC3 (Ref. 27) cells having a higher metastatic potential.^28^ This hypothesis needs to be tested in future work, but it could validate treatment planning algorithms based on the diameter of cancer cells from biopsy.^29^ Additionally, it may be advantageous to use the biopsy to create finite element models of multicellular clusters that account for cell packing density,^30^ as it has been shown that the electroporation threshold increases with higher cell packing density.^31^ These types of biopsy guided treatment planning procedures may alleviate concerns associated with patient-to-patient variability in response to therapy.

The significant correlation between the pre-treatment PSA and the change in current during IRE pulse delivery may be explained by the fact that there is also a significant correlation between the pre-treatment PSA and the ablation volume. Therefore, tumors with a higher pre-treatment PSA experienced a greater amount of electroporation spatially, which corresponds to a greater change in current. During IRE of the pancreas, researchers have shown that the change in tissue resistance was significant in predicting local failure or recurrence, but not overall disease free survival.^32^ For constant voltage electroporation systems, such as the NanoKnife, the change in current should be inversely proportional to the change in resistance (current = voltage/resistance). Also of interest is the fact that the change in PSA (pre-treatment minus post-treatment) was significantly correlated with the ablation volume. Lower post-treatment PSA scores have been associated with longer disease-free survival following high-intensity focused ultrasound.^33^

There are several limitations associated with the numerical models that can be attributed to either the nature of the clinical procedure or a lack of available data. As mentioned in Sec. IV, treatments were designed to completely destroy the tumor, and the boundary of the ablation volume extended into normal prostate tissues. Therefore, the reported electric field thresholds were likely influenced by healthy cells but provide at the least a conservative estimate for cancerous tissues. For a separate study to overcome this limitation, the tumor would need to be purposely undertreated, which may be possible in treat and resect procedures. Another limitation of this study is that the ablation volumes were measured at one week post-treatment using MRI. Tissue pathology is required to validate that the measurements are correlated with the volume of dead cells. It is possible that the ablation volume changes over time and an earlier or later time point would be more representative of the electric field threshold for cell death. Finally, major blood vessels,^34^ nerves, and ducts may distort the electric field.^20^ These heterogeneities should be included in future image reconstructions and simulations to study their impact on the IRE threshold.

## III. CONCLUSION

This investigation provides valuable data regarding the electric field threshold for IRE in human prostate cancer tissues. Volumetric reconstructions were created from one week post-treatment MRIs and incorporated into numerical models designed to simulate the electric field distribution during electroporation. The average electric field threshold that was determined (506 ± 66 V/cm) is comparable to that of other tissue types [e.g., normal porcine liver (423 V/cm),^17^ normal canine kidney (575 V/cm),^18^ normal canine brain (495–510 V/cm)^19^], as well as experiments performed on three-dimensional *in vitro* tumor models of pancreatic cancer (500 V/cm).^35^ It was also discovered that there is a potential correlation between the electric field threshold and the pre-treatment PSA, with higher PSA scores having a lower lethal threshold. This warrants future investigations into this relationship, as it opens the door for using a physiologic measurement to guide treatment planning.

## IV. METHODS

### A. Clinical workflow

This is a retrospective analysis of men who underwent therapeutic IRE, and no ID numbers are assigned to studies granted retrospectively according to local regulations. The results of previous clinical studies, which were performed with patients' consent, IRB approval, and Good Clinical Practices, can be found in Ref. 7. Briefly, patients were treated at Princess Grace Hospital in London/UK or St. Vincent's Prostate Cancer Centre in Sydney/Australia. All patients initially underwent a multi-parametric MRI in addition to demonstrating clinically significant prostate cancer through an analysis of their histology. Clinically significant cancer consists of a Gleason pattern ≥4 and/or a cancer core length ≥4 mm. Patients were treated while under general anesthesia with deep muscle paralysis using pancuronium bromide. A NanoKnife generator was used to deliver the IRE treatment. 4–6 needle electrodes were positioned not more than 2 cm apart transperineally at the margin of the cancer lesion under transrectal ultrasound guidance, and the electrode exposure length was set to either 1.5 cm or 2 cm depending on the size of the tumor. 90 pulses with a pulse length of 70 *μ*s were applied between each electrode pair. 10 test pulses were initially delivered in order to verify proper electrode placement and parameter selection. If the current was determined to be in the proper range (20–40 A), then the remaining 80 pulses were delivered. An example of the NanoKnife clinical workflow is shown in Fig. 1 for patient P2. The operator enters the electrode spacing using a graphical user interface, and the system calculates the required voltage to achieve a constant voltage-to-distance ratio of 1500 V/cm. The order of electrode pair activation is determined from the highest voltage (i.e., largest spacing) to the lowest voltage, and the remaining pulse parameters can be modified (pulse length and pulse number). The target volume was defined by MRI and histopathology with a safety margin of 3–5 mm. All treatments were performed within a single session. One week post-treatment, a contrast-enhanced MRI was performed to evaluate the local effect of the treatment. Additionally, PSA levels were evaluated every three months, and MRIs were repeated at six months and one year.

### B. IRE lesion reconstruction

Three-dimensional reconstructions of the ablation volume were created for 17 patients who participated in the clinical trial using the one week post-treatment MRIs. The ablation area is easily visible on post-treatment early scans as the non-enhancing zone on the contrast-enhanced MR sequence. Additionally, the locations of the electrodes and the apex and base of the prostate were labeled by the study clinicians. The location of the electrodes was determined by matching the grid reference recorded during the procedure and the margins of the ablation zone. It is important to note that the prostate gland (pre- and post-treatment) and tumor (pre-treatment) were also reconstructed but excluded from the simulations. This simplification was made for several reasons, including the fact that the ablation volume extended beyond the margin of the tumor into the prostate gland and a lack of available data on the differences in electrical properties between the tumor and the prostate gland.

Of the 17 reconstructions, 7 were omitted from the analysis for various reasons. 2 patients exhibited large protrusions in their ablation volume which might have resulted from underlying heterogeneous features (e.g., urethra, bowel, and neurovascular bundle) not included in the simulation; 3 patients exhibited non-contiguous ablations; 2 patients received an extra treatment for which the electrode marker data were unavailable. The 10 patients who were analyzed all received an equivalent energy dose. 9 of the patients received 90 pulses per treatment with a pulse duration of 70 *μ*s, and 1 patient (JC01) received 70 pulses per treatment with a pulse duration of 90 *μ*s. As mentioned, based on the electrode spacing, the voltage-to-distance ratio was 1500 V/cm. An exception was patient P1, who had one treatment at 1364 V/cm. In this case, the electrode spacing (2.2 cm) required a larger voltage than available on the NanoKnife (3000 V maximum) to reach 1500 V/cm (see Table I).

### C. Numerical simulations

A 3D finite element model was developed using COMSOL Multiphysics (version 5.2, Burlington, MA) to calculate the electric field distribution during IRE therapy. This methodology has been validated as a means to predict the electric field threshold for reversible electroporation and IRE.^36^ The ablation volume was placed inside a cube (7.5 cm), which was large enough to encompass the surrounding prostate gland (Fig. 2), and the four electrodes were modeled as stainless steel cylinders [*σ* = 2 × 10^6^ S/m (Ref. 37)] with a height set according to the exposure length used during treatment. Initially, the electrical conductivity of the tissue (ablation volume and cube) was set to a static, baseline value (*σ*_0_ = 0.284 S/m), which was determined by low-voltage (50 V) pre-pulse current measurements in normal canine prostate.^20^ It was not possible to perform similar measurements from the human clinical data included herein, as the NanoKnife generator does not report pre-pulse currents, and the voltage delivered during the first pulse was high enough to induce electroporation. Boundary conditions (electric potential and ground) were applied to the outer surfaces of electrode pairs to mimic the clinical procedure. The electric field distribution within the tissue (E=−∇ϕ) was obtained for each electrode pair by solving

$$\nabla \cdot \left(\sigma_{0}\nabla \phi \right)=0,$$ (1)

where ϕ is the electric potential. The overall electric field distribution from all six treatments was defined as a separate variable by taking the maximum values from each pair-based combination. Then, the electric field threshold was calculated by performing a volume integration of the maximum electric field distribution above a certain electric field contour. The value of the field contour was varied in 1 V/cm increments until the volume matched the actual ablation volume.

A second set of simulations were performed with a dynamic electrical conductivity function to account for the electroporation-induced conductivity increase from the formation of nanopores

$$\nabla \cdot \left(\sigma (\left|E\right|)\nabla \phi \right)=0.$$ (2)

This has been shown to be more accurate than the static model in predicting the permeabilized volume of tissues.^38^ The conductivity function, *σ*(|*E*|), was defined as a step function that increased from *σ*_0_ to *σ*_max_ over a transition zone of 800 V/cm centered at 500 V/cm with a continuous second derivative (Fig. 3). The characteristics of the transition zone were chosen to mimic the function developed for normal canine prostate.^20^ A parametric study was run on *σ*_max_ for each patient to match the calculated current to the maximum measured current during the last set of ten pulses from the first electrode pair activation (see Fig. 1). The first electrode pair was evaluated to avoid compounding effects from multiple treatments. The current was calculated by integrating the normal current density across a cut plane between the electrodes. Following the determination of the patient-specific conductivity function, the solution for the electric field distribution was obtained under these conditions for each electrode pair combination. Then, an additional variable was created for the overall electric conductivity distribution from all six treatments by taking the maximum values from each pair-based simulation. Finally, the electric field threshold was calculated in the same fashion as described earlier.

### D. Statistical analysis

Correlations were determined between all variables of interest, including the pre-treatment PSA, change in current during treatment, change in conductivity during treatment, and the electric field threshold. Additionally, the correlation between the ablation volume and the change in PSA was also determined. All statistical analyses were conducted using JMP Pro 11 (Cary, North Carolina, USA) with a significance level of *p* ≤ 0.05. Due to a low sample size (*n* = 10), the statistical power for most relationships was very low (<60%). Despite this low power, significance relationships were still found.
